# An unusual association of type II Mayer-Rokitansky-Kuster-Hauser syndrome, turner mosaic syndrome and tubo-ovarian inguinal hernia– case report and review of literature

**DOI:** 10.1186/s13048-022-01067-0

**Published:** 2023-02-23

**Authors:** Reeta Mahey, Anubhuti Rana, Rohitha Cheluvaraju, Surabhi Vyas, Ritu Raj, Neerja Bhatla

**Affiliations:** Department of Obstetrics and Gynaecology, AIIMS, New Delhi, India

**Keywords:** Inguinal hernia, Turner syndrome, Amenorrhoea, Ectopic ovary, Mullerian agenesis

## Abstract

**Background:**

Herniation of ovaries and Mullerian structures into inguinal canal is usually reported in infants and is rare among adults. We are presenting a rare case of Mullerian agenesis and Turner mosaic syndrome with tubo-ovarian inguinal hernia.

**Case presentation:**

A 17-year-old girl presented with complaints of primary amenorrhea, phenotypical features of Turner syndrome with left inguinal hernia and severe hypertension. Baseline hormonal analysis was normal. Karyotype revealed Turner mosaic with 46XX (85%); 45XO (15%). MRI showed Mullerian agenesis with normally located right ovary in pelvis and left ovary prolapsed through deep inguinal ring into the canal of Nuck. Anti-hypertensives were started and patient optimized for surgery. Laparoscopic hernia repair and repositioning of left ovary into the pelvis was done. Patient had uneventful post-operative course and was discharged in stable condition on anti-hypertensive medication. Future reproductive issues and need of passive vaginal dilatation or vaginoplasty before marriage were explained to the patient and family.

**Conclusion:**

This is the first ever reported case with unusual association of atypical MRKH, Turner mosaic syndrome and tubo-ovarian hernia into the inguinal canal. The case emphasizes the need and importance of complete work up of these atypical cases as patients may have more than one cause of primary amenorrhea and complete evaluation is must before any medical or surgical intervention.

## Introduction

Herniation of uterus and adnexa have been reported in up to 31% indirect hernial sacs among infant girls which is due to incomplete closure of the canal of Nuck. The incidence decreases with age and is very rare among adolescent and adult females [1]. Most of these cases are associated with congenital anomalies of the female genital tract.

Mayer-Rokitansky-Kuster-Hauser syndrome (MRKHS) is characterized by female phenotype, primary amenorrhea, normal secondary sexual characteristics, absent vagina, absent or rudimentary uterus and normally developed ovaries. Incidence of MRKHS is 1 in 4000–5000 females [2]. The syndrome is of two types: Typical – Isolated uterovaginal agenesis, and atypical – Mullerian agenesis along with malformations of kidneys or ovaries, including MURCS (Mullerian aplasia, renal aplasia, and cervicothoracic somite dysplasia) which is a severe variant of atypical MRKH along with skeletal, heart, muscular weakness and anomalies in auditory system [3]. Exact incidence of MRKHS with genital inguinal hernia is very rare and probably under-reported [4].

Turner syndrome or gonadal dysgenesis in females is characterized by complete or partial absence of second sex chromosome with or without cell line mosaicism [5]. Overall incidence is 1:2500 live birth females. These females have absent or insufficient development of ovaries and may present with lack of development of secondary sexual characteristics, primary amenorrhea and/or specific Turner stigmata. Karyotype may reveal 45XO, mosaicism or absence of some specific part on X chromosome [6].

The association of Mullerian agenesis and gonadal dysgenesis, though reported, is exceedingly rare [7]. We report an unusual case of type II Mayer-Rokitansky-Kuster-Hauser syndrome (MRKHS) with mosaic Turner syndrome associated with utero-tubo-ovarian hernia into inguinal canal.

## Case

A 17-year-old girl, not sexually active presented to the Gynaecology outpatient department with chief complain of primary amenorrhea. There was no history of cyclic abdominal pain, excessive hair growth, weight gain/loss or galactorrhea. She was studied till grade 6 with otherwise normal intelligence. There was no remarkable past history. Her two younger sisters (13 years and 12 years) had normal secondary sexual characteristics but not yet attained menarche.

General physical examination revealed short height (134 cm), weight 34 kg, BMI-18.9 kg/m^2^, webbed neck along with an increased carrying angle of the elbow. Breast and pubic hair were Tanner 3 and axillary hair were present. Vitals were within normal limits except that her blood pressure was 200/120 mmHg. Systemic examination including cardiovascular and respiratory systems and abdominal examination were within normal limits. On local examination, a 3 × 3 cm firm, non-tender swelling was noticed in left inguinal region. The swelling had smooth and regular margins and there was no redness or local rise of temperature. Cough impulse was present and swelling was irreducible. Right inguinal region was normal. External genitalia were normal; however, examination revealed a blind vagina with no uterus felt. Per-rectal examination confirmed the same. On enquiring about the swelling in left groin, mother revealed that she had noticed this swelling when the patient was of 2 years and was initially of pea size. This swelling had gradually increased to the present size of a small lemon and was not associated with pain or dragging sensation.

**Fig. 1:**
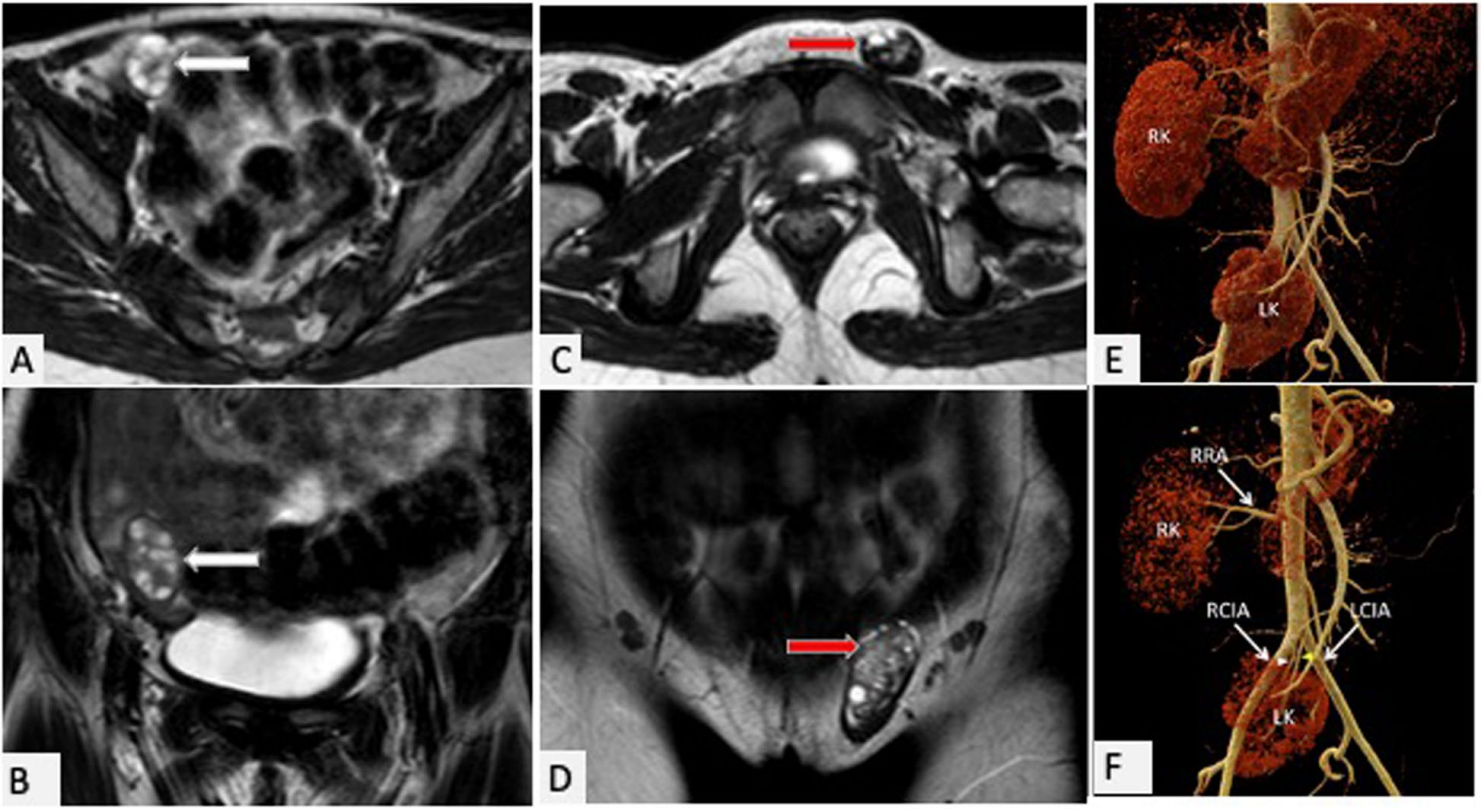
MRI images (**A**, **B**) showing axial and coronal T2w images showing anteriorly located right ovary (white arrows) at the internal inguinal ring. MRI images (**C**, **D**) showing axial and coronal T2w images showing left ovary (red arrows) located in the left inguinal canal. Volume rendered images of computed tomography angiography (**E** and **F**) show the left kidney (LK) in the pelvis with two branches (indicated by white and yellow arrowheads respectively) arising from the right common iliac artery (RCIA) and the left common iliac artery (LCIA) respectively seen supplying the ectopic left kidney. The right kidney (RK) was normally located with a normal right renal artery (RRA)

**Fig. 2:**
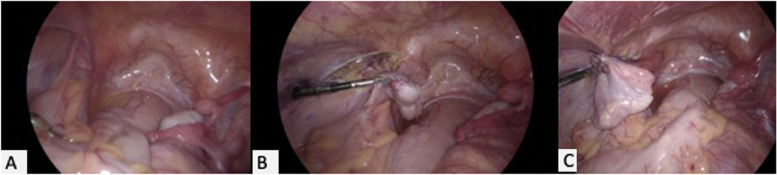
**A** Laparoscopy showing normally located right uterine nodule, tube and ovary and left side ovary and Mullerian structures prolapsed into inguinal canal. **B** Deep inguinal ring excised and left uterine bud, fallopian tube and ovary pulled out from the inguinal canal into the pelvis. **C**-Left ovary fixed to the peritoneum after closing the deep inguinal ring

With a clinical provisional diagnosis of Turner syndrome in view of primary amenorrhea, short height, severe hypertension, patient was admitted for work up and further evaluation. Baseline hormonal profile showed FSH 3.9 mIU/ml (normal range-2.8-11.3 mIU/mL); LH 13.3 mIU/ml (normal range for follicular phase 1.1–11.6 mIU/mL, for luteal phase 0–14.7 mIU/mL); TSH-1.15 uIU/ml; prolactin- 14.1 ng/ml; AMH-10.22 ng/ml; testosterone-24.4 ng/dl; E2–49.21 pg/ml. Karyotype analysis revealed Turner mosaic with 46XX (85%); 45XO (15%). MRI abdomen and pelvis revealed a hypoplastic uterine nodule on right side with normally located ovary; left ovary herniated in the left indirect inguinal hernial sac; and malrotated and ectopic left kidney (Fig. 1A-D). Echocardiography and CT angiography (Fig. 1E and F) revealed concentric left ventricular hypertrophy and malrotated and ectopic left kidney. After reconfirming the karyotype and imaging, decision for reposition of left ovary was taken after thorough counselling of the patient and family. Laparoscopic inguinal hernia repair was done and Mullerian structures (small uterine bud, fallopian tube) and ovary were pulled out of the left deep inguinal ring; deep inguinal ring was closed and ovary was fixed to the peritoneum (Fig. 2). Patient had uneventful post-operative course and was discharged in stable condition after 2 days on anti-hypertensives. Family has been counselled about the condition and need of passive vaginal dilatation or vaginoplasty before marriage.

## Discussion

The present case describes first ever reported association of Type II MRKHS and Turner mosaic syndrome along with tubo-ovarian inguinal hernia to the best of our knowledge.

Inguinal hernia of the ovary and fallopian tube is commonly seen in infancy and present within initial 2 years of age [8]. Short inguinal canal, oblique direction of canal in abdominal wall and diverticulum of Nuck are various anatomical conditions which predispose to adnexal entrapment and subsequent inguinal hernia of the Mullerian structures. Various theories postulated for this include non-fusion of the Mullerian ducts leading to hypermobility of the ovary or congenitally elongated ovarian ligaments and patent processus vaginalis (also known as canal of Nuck) which should have been closed by 1 year of post-natal life [9]. Ovarian torsion and infarction have been reported in 2–37% of these cases. Hence herniorrhaphy and reposition of ovary should be considered in all cases including asymptomatic ones [10]. This is essential to preserve ovarian function and may be done through either open or laparoscopic approach.

The exact association of MRKHS with indirect inguinal hernia of ovaries/Mullerian structures is not clear due to under-reporting of most of the cases. Table 1 describes the cases of MRKHS along with ovarian inguinal hernia. This association is often discovered incidentally through diagnostic imaging when patients present with local pain arising from a hernia, primary amenorrhoea or even later in life with infertility issues. In our case, it was detected as while patient presented with primary amenorrhea and examination revealed inguinal swelling.

**Table 1:** Reports of MRKHS and Ovarian inguinal hernia published till date

Author	Age (yr)	Chief complaint/s	Diagnosis	Karyotype		Management
Elliot DC et al. (1989) [11]	19	Mass in left inguinal region × 2 years Pain and left groin bulge × 2 days,	Unicornuate uterus; inguinal hernia of fallopian tube, ovary and rudimentary uterine horn	46, XX	Left pelvic kidney	Surgical exploration of left groin and resection of ovary, fallopian tube and rudimentary horn of uterus (Inguinal approach)
Kriplani A et al. (2000) [12]	NA	Primary amenorrhea,	Mullerian agenesis with inguinal hernia of left horn of uterus and ovary	46, XX		NA
Bazi et al. 2006 [13]	12	Cyclic alternating inguinal pain	Mullerian agenesis with bilateral inguinal ovaries	46, XX	Left kidney seen at the level of right iliac fossa (crossed ectopia	Surgery suggested but deferred on patient’s wishes and started on low dose OCP to prevent cyclic inguinal pain
Omari et al. (2011) [4]	31	Primary amenorrhea, infertility and dyspareunia	MRKH with utero-ovarian inguinal hernia	46, XX	Single right pelvic kidney	Surgical exploration and reduction of hernia (Inguinal approach) + William vaginoplasty
Demirel et al. (2012) [14]	10	Incidentally detected ovary in hernial sac during herniorrhaphy	MRKH type II with left ovarian hernia	46, XX	Absent left kidney; cervical hemi-vertebra	Surgical exploration and reduction of hernia
Palepu et al. (2015) [15]	20	Primary amenorrhea, bilateral groin swellings, acute onset of right-side pain	MRKH, bilateral tubo-ovarian hernia and right-side ovarian torsion	46XX		laparotomy, herniorrhaphy, ovarian biopsy and repositioning of ovaries.
Mohanty et al. (2017) [16]	20	Primary amenorrhea	Mullerian agenesis (Type 1) with bilateral ovarian inguinal hernia	46, XX	Bilateral ureteroceles	Not given details about surgery
Verma R et al. (2018) [17]	45	Pain in inguinal area (started at age 43), primary amenorrhea	MRKHS	NA	Left kidney absent	Surgical exploration for resection of Mullerian duct remnants and hernia repair (Inguinal approach)
Khan et al. (2019) [18]	18	Primary amenorrhea, swelling in left groin × 6 months,	MRKH	NA	Left renal agenesis	Laparoscopic total extraperitoneal repair of the ovarian inguinal hernia
Jafari et al., (2020) [19]	13	Primary amenorrhea, swelling and pain in left groin	MRKHS, left inguinal hernia of ovary, fallopian tube and rudimentary horn of bicornuate uterus,	NA	Ectopia renal of left lower quadrant	Inguinal approach, repositioning of structures in pelvis and hernia repair
Saini et al. [20]	20	Right inguinal swelling since 2 years of age, primary amenorrhea	MRKHS, unilateral lung agenesis, right inguinal ovarian hernia	46, XX	Absent right kidney, fusion of posterior elements of 2nd- 7th cervical vertebral bodies; scoliosis	Inguinal approach, repositioning of structures in the pelvis and hernia repair
Present Case	17	Primary amenorrhea, swelling in inguinal region since 2 years of age	MRKH, Turner’s Mosaic, inguinal hernia of left ovary and fallopian tube	46XX (85%); 45XO (15%)	Ectopic and mal-rotated left kidney	Laparoscopic ovarian reposition and repair of hernial sac

Our case emphasizes the importance of complete work up, detailed examination, and hormone analysis in these patients. The clinical features did not fit into any one disorder as patient had features of Turner syndrome (short height, webbed neck, severe hypertension); MRKHS (primary amenorrhea, normal secondary sexual characteristics, blind vagina, absent uterus); and androgen insensitivity syndrome (primary amenorrhea, blind vagina, absent uterus and inguinal mass).

Karyotype testing in these atypical cases is the most important and crucial investigation before clinching the final diagnosis. The indication of karyotype testing in MRKHS is to differentiate it from androgen insensitivity syndrome or if patient has clinical findings not fitting into diagnosis of MRKHS. Also, in patients presenting with inguinal masses, the importance of karyotype can’t be ignored as inguinal mass may be either testis or undifferentiated gonads with Y-line component. Magnetic Resonance Imaging (MRI) is essential to delineate the anatomy of renal and reproductive organs including uterus, fallopian tubes, exact location of ovaries, anatomy of contents of inguinal canal. This will also help to counsel the patient and family, extent and route of surgery, decision to preserve the ovary/contents of inguinal canal. Especially in cases with Turner mosaic, ruling out Y-line germ cells is very important as presence of Y-line will warrant gonadectomy.

Early diagnosis and timely management are the key as ovary is prone to torsion and infarction in the inguinal canal. In a recent systematic search of literature of 17 cases of ovarian hernia were identified in reproductive aged women, 15 of which underwent an emergency surgery as they presented with tender irreducible lump. The most common surgery performed included repositioning of structures with repair of hernia, however, oophorectomy and salpingo-oophorectomy was required in 2 cases each respectively [21]. Early identification of inguinal hernia and prompt surgical intervention plays a vital role in management of such cases as the prolapsed ovary may undergo torsion, incarceration and may need surgical removal if vitality is lost. In asymptomatic females which may remain undiagnosed, ectopic location of ovaries itself might be predisposing factor for early ovarian ageing and malignancies. Our patient did not require oophorectomy /salpingo-oophorectomy and was managed by laparoscopic repair of the hernia along with repositioning of the structures.

Although karyotype is normal in typical MRKH syndrome, rarely cases have been reported of association if MRKHS with different types of gonadal dysgenesis. In a recent case report of MRKHS and Turner syndrome, the authors reviewed the literature and their case was the 26th ever reported case of association of MRKH and Turner syndrome [6]. Our case is the 27th showing association of Mullerian agenesis and Turner syndrome.

Indications of echocardiography include severe hypertension as in the present case to rule out cardiac dysfunction and renal vascular anomalies especially renal artery stenosis. Our case had ectopic and mal-rotated left kidney and getting blood supply from left and right common iliac arteries. These investigations are warranted in females with Turner syndrome.

Exact reason for unusual association of Mullerian agenesis and gonadal dysgenesis is not clear and needs further exploring the genetic basis of these rare combinations. These patients are at risk of hypertension, osteoporosis and premature ovarian insufficiency/ ovarian failure. Long term hormone replacement therapy may be required but the problem of infertility remains unsolved due to both ovarian factor and uterine factor (Mullerian agenesis). Sexual function can be provided either by passive vaginal dilatation or vaginoplasty as per patient choice. Psychological counselling of patient and family and long-term follow-up should be combined with routine medical care of these patients.

## Conclusion

To conclude we report an unusual and rare association of MRKHS and Turner mosaic syndrome along with tubo-ovarian inguinal hernia which presented at age of 17 years. The case emphasizes the need of complete work up, importance of detailed clinical examination, karyotype and imaging in females presenting with primary amenorrhea and specific clinical findings. Patient may simultaneously have two independent causes of primary amenorrhea. Although rare, MRKHS, Turners syndrome and tubo-ovarian inguinal hernia can coexist in the same patient which would necessitate extensive counseling for future implications on fertility, pregnancy and quality of life. Laparoscopic approach helped early post-operative recovery in this case.
